# Molecular and morphological characterization of the amaryllis lesion nematode, *Pratylenchus hippeastri* (Inserra et al., 2007), from California

**DOI:** 10.21307/jofnem-2020-058

**Published:** 2020-07-06

**Authors:** Zafar A. Handoo, Andrea M. Skantar, Mihail R. Kantor, Saad L. Hafez, Maria N. Hult

**Affiliations:** 1 Mycology and Nematology Genetic Diversity and Biology Laboratory, USDA, ARS, Northeast Area, Beltsville, MD 20705; 2 University of Idaho, Parma, ID 83660

**Keywords:** Grapevine, Lesion nematode, Molecular markers, *Pratylenchus hispeastri*

## Abstract

Root-lesion nematodes (*Pratylenchus* spp.) are among the most important nematode pests on grapevine along with root-knot, dagger, and ring nematodes. In 2019, two samples of both soil and roots were collected from a vineyard in Delano, Kern County, California and submitted to the United States Department of Agriculture, ARS, Mycology and Nematology Genetic Diversity and Biology Laboratory, Beltsville, MD, for identification purposes. Females and juveniles of *Pratylenchus sp.* were recovered from the root and soil samples using the sugar centrifugal flotation and Baermann funnel extraction methods. Both morphological observations and molecular analysis of internal transcribed spacer (ITS), 28S large subunit ribosomal DNA, and mitochondrial cytochrome oxidase (COI) sequences indicated that the specimens recovered from the soil and roots were *Pratylenchus hippeastri*. To the best of the authors’ knowledge, this is the first report of *P. hippeastri* from California including the first record of this species on grapevine and the second state record in North America. Damages caused by nematodes cannot be over-emphasized, although economic importance of *P. hippeastri* has never been established. Hence, there is an urgent need to investigate the economic impact of this nematode in vineyards in California State in order to develop sustainable management strategies.

Grapevine (*Vitis vinifera*) is one of the most extensive fruit crops of agricultural system worldwide (Torregrosa et al., 2015). As per United States Department of Agriculture, National Agricultural Statistics Service, the United States grape production in 2017 was 7,363,260 tons. The highest acreage planted with grapevine is in California, with a total of 880,000 acres planted in 2017 and 925,000 in 2018 (USDA, National Agricultural Statistics Service, 2019). The genus *Pratylenchus* Filipjev, 1936 contains approximately 100 species (Geraert, 2013; Qing et al., 2019), with new species being described very frequently. Root-lesion nematodes are among the most prevalent nematodes that can infect and cause damage to the grapevine roots (Téliz et al., 2007; Howland et al., 2014). The large number of species as well as the vast number of hosts makes this genus very important from an economic perspective. *Pratylenchus hippeastri*, also known as the amaryllis lesion nematode, has been previously reported only from Florida (Gozel et al., 2007; DeLuca et al., 2010), China (Wang et al., 2016) and more recently from South Africa (Shokoohi, 2019; Knoetze et al., 2019). Currently, the host range of this nematode is narrow, being reported only on three hosts, amaryllis, bromeliads, and apples (Gozel et al., 2007; DeLuca et al., 2010; Wang et al., 2016; Knoetze et al., 2019) and from the rhizosphere around Cape Willow trees, *Salix mucronata* (Shokoohi, 2019). This report represents the first detection of this species on grapevines in California, thus representing the second report of this nematode in North America.

## Materials and methods

Two soil samples and grapevine roots were sent to the Mycology and Nematology Genetic Diversity and Biology Laboratory, Beltsville, MD in 2019. The origin of the soil samples was a vineyard from Mosca, Alamosa County, Co. Nematodes were extracted from soil using sugar centrifugal flotation and Baermann funnel methods.

Nematodes were fixed in 3% formaldehyde and processed to glycerin by the formalin glycerin method (Golden, 1990; Hooper, 1970). Photomicrographs of the specimens were made with a Nikon Eclipse Ni compound microscope using a Nikon DS-Ri2 camera. Measurements were made with an ocular micrometer on a Leica WILD MPS48, Leitz DMRB compound microscope. All measurements are in micrometers unless otherwise stated.

The molecular identification was performed using DNA extracted from single nematodes as template in PCR reactions. The internal transcribed spacer (ITS) 1 & 2 rDNA region was amplified with primers TW81 [5’-GTTTCCGTAGGTGAACCTGC-3’] and AB28 [5’-ATATGCTTAAGTTCAGCGGGT-3’] (Skantar et al., 2012), producing a PCR amplicon of 964 bp. The PCR product was cleaned with the Monarch DNA Gel Extraction Kit (NEB, Ipswitch, MA) and then cloned using the Strataclone PCR Cloning Kit (Agilent, Santa Clara, CA). Cloned plasmid DNA was prepared with the Monarch Plasmid Miniprep Kit (NEB) and sequenced by Genewiz, Inc. Mitochondrial cytochrome oxidase I (COI) was amplified with JB3 [5’-TTTTTTGGGCATCCTGAGGTTTAT-3’] and JB5 [5’-AGCACCTAAACTTAA AACATAATGAAAATG-3’] (Derycke et al., 2005) as described in Ozbayrak et al. (2019). PCR amplicons of 403 bp were cleaned and sequenced directly with the same primers. The 28 S large ribosomal subunit D2-D3 expansion segment was obtained via amplification with the primers D2A [5’-ACAAGTACCGTGAGGGAAAGTTG-3’] and D3B [5’-TCGGAAGGAACCAGCTACTA-3’] (De Ley et al., 2005; Ye et al., 2007), producing sequences of 737 to 761 bp using the same primers. Raw sequence reads were processed in Sequencher 5.4.6 (Genecodes, Inc., Ann Arbor, MI). GenBank accession numbers for newly obtained sequences were assigned as follows: ITS rDNA (MT090056), COI (MT093835-MT093837), and 28 S rDNA (MT090067-MT090067). Selected sequences from *P. hippeastri* and other species were obtained from GenBank.

DNA sequences were analyzed by BlastN to identify similarity to those in GenBank. Evaluations of intraspecific and interspecific variation were conducted using sequence alignment algorithms within Geneious Prime 2020.1.0). Phylogenetic analysis was conducted by Bayesian Inference (Huelsenbeck and Ronquist, 2001) via the CIPRES Gateway (Miller et al., 2010) plug-in in Geneious. For COI sequence alignments, the model of nucleotide evolution was determined with jModelTest 2.1.7 (Darriba et al., 2012) to be GTR + I + G, according to Akaike Information Criteria (AIC). Bayesian analysis was run with random starting trees, four chains for 2 × 10^6^ generations, with Markov chains sampled every 500 generations. Two runs were performed for each analysis. Burn-in samples were discarded, and convergence was evaluated, with remaining samples retained for further analysis. Topologies were used to generate 50% majority rule consensus tree with posterior probabilities greater than 0.5 shown on appropriate clades.

### Description

#### Measurements

In females (*n* = 10): body length (mean = 436.0 μm, range = 402.0-476.0 μm), stylet (15.0, 13.0-15.5), body width (20.0, 15.0-28.5), head end to posterior end of esophageal glands (104.0, 98.0-111.0), anal body width (12.0, 10.5-13.0), tail length (26.0, 21.0-29.0), a (25.0, 20.0-32.0), b (4.2, 3.6-4.7), c (17.0, 15.0-21.0), c’ (2.1, 1.6-2.5) and V (77.0%, 74.0-79.0%). Four lines are present in the lateral field.

The morphometric details of females were recorded and compared to closely related species which were consistent with *Pratylenchus hippeastri* (Inserra et al., 2007).

#### Molecular analysis

**Figure 1:**
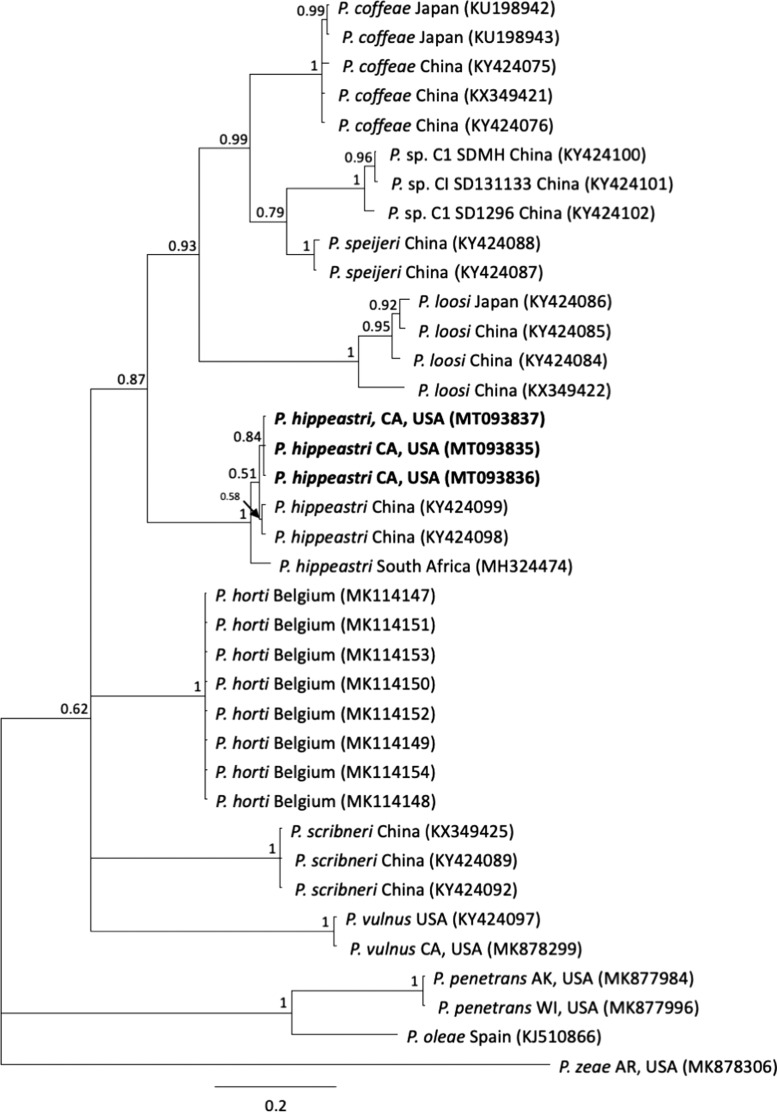
Phylogenetic relationships of *Pratylenchus hippeastri* and other selected lesion nematodes, as inferred from a 402 bp alignment of mitochondrial COI DNA sequences, with *P. zeae* as the outgroup. A 50% majority rule consensus tree obtained from Bayesian analysis was generated using the GTR + I + G model of nucleotide substitution. Branch support values above 50% are shown on appropriate branches. New sequences are highlighted in bold.

Molecular identification of the California population as *P. hippeastri* was confirmed by BlastN comparison of multiple ribosomal and mitochondrial markers to available GenBank sequences. The 28 S rDNA sequences were > 99.8% similar (differing at 0-4 bp) to several isolates of *P. hippeastri*, including those from amaryllis in Florida (DQ498829) and Israel (KJ001715), apple from South Africa (MK749422) and China (KR029084), bromeliads (FN994114, FN55480) and bottlebrush (GU131130) from Florida, and ornamental trees from Florida (GU131127), Japan (KC796703; KP161608; KP161609), and South Africa (MH324472). The ITS rDNA sequence was 99.9% similar to several *P. hippeastri* sequences, including populations from the USA (Florida) (FN5544888), Israel (KJ001718), Japan (KC796701), China (FJ712932), and South Africa (MH324471). Phylogenetic trees inferred from alignments of either 28 S or ITS rDNA placed the California population within the highly supported monophyletic group of *P. hippeastri*, nearest to *P. floridensis* and *P. parafloridensis* (not shown), in agreement with prior studies (Subbotin et al., 2008; DeLuca et al., 2010; Wang et al., 2016; Shokoohi, 2019). Mitochondrial COI sequences showed 99% identity to those from China (host not reported; KY424099) and those isolated from the rhizosphere soil samples of Cape Willow trees (*Salix mucronata*) in the North-West Province, South Africa (MH324474). These sequences and those of selected other *Pratylenchus* species were assembled into an alignment of 402 bp for phylogenetic analysis by Bayesian Inference (Figure 1). According to these results, the California population clustered more closely with the Chinese population (0-4 bp differences) than with the South African population (11-12 bp different). The placement is consistent with the results of Shokoohi (2019). No COI sequences were available for *P. floridensis* or *P. parafloridensis*, so the *P. hippeastri* group grouped nearest to *P. loosi.*

Based upon the unambiguous similarity of all examined DNA markers with those previously reported for the species by several authors (Gozel et al., 2007; Subbotin et al., 2008; DeLuca et al., 2010; Wang et al., 2016; Knoetze et al., 2019; Shokoohi, 2019) and the morphological data by Inserra et al. (2007), we identify this isolate as *Pratylenchus hippeastri* (Inserra et al., 2007). To our knowledge this represents the first report of the amaryllis lesion nematode (*Pratylenchus hippeastri*) in California as well as the first report on grapevine.

## References

[ref001] Darriba D., Taboada G., Doallo R. and Posada D. 2012 jModelTest 2: more models, new heuristics and parallel computing. Nature Methods 9:772. 10.1038/nmeth.2109 PMC4594756 22847109

[ref002] De Ley P., Tandingan De Ley I., Morris K., Abebe E., Mundo-Ocampo M., Yoder M., Heras J., Waumann D., Rocha-Olivares A., Burr A. H. J., Baldwin J. G. and Thomas W. K. 2005 An integrated approach to fast and informative morphological vouchering of nematodes for applications in molecular barcoding. Philosophical Transactions of the Royal Society B 360:1945–1958. 10.1098/rstb.2005.1726 PMC1609217 16214752

[ref003] DeLuca F., Troccoli A., Duncan L. W., Subbotin S. A., Waeyenberge L., Moens M. and Inserra R. N. 2010 Characterisation of a population of *Pratylenchus hippeastri* from bromeliads and description of two related new species, *P. floridensis* n. sp. and *P. parafloridensis* n. sp., from grasses in Florida. Nematology 12:847–868.

[ref004] Derycke S., Remerie T., Vierstraete A., Backeljau T., Vanfleteren J., Vincx M. and Moens T. 2005 Mitochondrial DNA variation and cryptic speciation within the free-living marine nematode Pellioditis marina. Marine Ecology-Progress Series 300:91–103.

[ref005] Geraert E. 2013 The Pratylenchidae of the World: Identification of the Family Pratylenchidae (Nematoda: Tylenchida) Academia Press, Gent, 430pp.

[ref006] Golden A. M. 1990 “Preparation and mounting nematodes for microscopic observations”, in Zuckerman B. M., Mai W. F. and Krusberg L. R. (Eds), Plant Nematology Laboratory Manual University of Massachusetts Agricultural Experiment Station, Amherst, MA 197–205.

[ref007] Gozel U., Duncan L., Inserra R., Bernard E., Dunn D. and Troccoli A. 2007 *Pratylenchus hippeastri* n. sp. (Nematoda: Pratylenchidae) from amaryllis in Florida with notes on *P. scribneri* and *P. hexincisus* . Nematology 9:25–42.

[ref008] Hooper D. J. 1970 Handling, fixing, staining and mounting nematodes. Technical Bulletin. Ministry of Agriculture, Fisheries and Food, (5th ed., 2), 39–54.

[ref009] Howland A. D., Schreiner R. P. and Zasada I. A. 2014 Spatial distribution of plant-parasitic nematodes in semi-arid *Vitis vinifera* vineyards in Washington. Journal of Nematology 46:321. 25580024 PMC4284083

[ref010] Huelsenbeck J. P. and Ronquist F. 2001 MRBAYES: Bayesian inference of phylogenetic trees. Bioinformatics 17:754–755. 11524383 10.1093/bioinformatics/17.8.754

[ref011] Inserra R. N., Troccoli A., Gozel U., Bernard E. C., Dunn D. and Duncan L. 2007 *Pratylenchus hippeastri* n. sp. (Nematoda: Pratylenchidae) from amaryllis in Florida with notes on *P. scribneri* and *P. hexincisus* . Nematology 9:25–52.

[ref012] Knoetze R., van den Berg E., Girgan C. and van der Walt L. 2019 First report of the root lesion nematode, *Pratylenchus hippeastri*, on apple in South Africa. Journal of Plant Diseases and Protection 126:607–609.

[ref013] Miller M. A., Pfeiffer W. and Schwartz T. 2010 Creating the CIPRES Science Gateway for inference of large phylogenetic trees. Proceedings of the Gateway Computing Environments Workshop (GCE), New Orleans, November 14, LA, 1–8.

[ref014] Ozbayrak M., Todd T., Harris T., Higgins R., Powers K., Mullin P., Sutton L. and Powers T. 2019 A COI DNA barcoding survey of *Pratylenchus* species in the Great Plains Region of North America. Journal of Nematology 51:1–21. 10.21307/jofnem-2021-100 PMC8662978 34901874

[ref015] Qing X., Bert W., Gamliel A., Bucki P., Duvrinin S., Alon T. and Braun Miyara S. 2019 Phylogeography and molecular species delimitation of *Pratylenchus capsici* n. sp., a new root lesion nematode in Israel on pepper (*Capsicum annuum*). Phytopathology 109:847–58. 30422745 10.1094/PHYTO-09-18-0324-R

[ref016] Shokoohi E. 2019 New data on known species of *Hirschmanniella* and *Pratylenchus* (Rhabditida, Pratylenchidae) from Iran and South Africa. Journal of Nematology 51:1–26. 10.21307/jofnem-2019-041 PMC6909020 34179797

[ref017] Skantar A. M., Handoo Z. A., Zanakis G. N. and Tzortzakakis E. A. 2012 Molecular and morphological characterization of the corn cyst nematode, *Heterodera zeae*, from Greece. Journal of Nematology 44:58–66. 23482617 PMC3593258

[ref018] Subbotin S. A., Ragsdale E. J., Mullens T., Roberts P. A., Mundo-Ocampo M. and Baldwin J. G., 2008 A phylogenetic framework for root lesion nematodes of the genus *Pratylenchus* (Nematoda): Evidence from 18S and D2–D3 expansion segments of 28S ribosomal RNA genes and morphological characters. Molecular Phylogenetics and Evolution 48:491–505. 18514550 10.1016/j.ympev.2008.04.028

[ref019] Téliz D., Landa B. B., Rapoport H. F., Camacho F. P., Jiménez-Díaz R. M. and Castillo P. 2007 Plant-parasitic nematodes infecting grapevine in southern Spain and susceptible reaction to root-knot nematodes of rootstocks reported as moderately resistant. Plant Disease 91:1147–1154. 30780656 10.1094/PDIS-91-9-1147

[ref020] Torregrosa L., Vialet S., Adivèze A., Iocco-Corena P. and Thomas M. R. 2015 “Grapevine (*Vitis vinifera* L.)”, in Wang K. (Ed.), Agrobacterium Protocols Springer, New York, NY, 177–194. 10.1007/978-1-4939-1658-0_15 25416258

[ref021] USDA, National Agricultural Statistics Service (2019), California Field Office. Grape reports, available at: https://www.nass.usda.gov/Statistics_by_State/California/Publications/Specialty_and_Other_Releases/Grapes/Acreage/2019/201904grpacSUMMARY2018Crop.pdf (accessed March 27, 2020).

[ref023] Wang H. H., Zhuo K. and Liao J. L. 2016 Morphological and molecular characterization of *Pratylenchus hippeastri*, a new record of root lesion nematode associated with apple in China. Pakistan Journal of Zoology 48:665–671.

[ref024] Ye W., Giblin-Davis R. M., Davies K. A., Purcell M. F., Scheffer S. J., Taylor G. S., Center T. D., Morris K. and Thomas W. K. 2007 Molecular phylogenetics and the evolution of host plant associations in the nematode genus Fergusobia (Tylenchida: Fergusobiinae). Molecular Phylogenetics and Evolution 45:123–141. 17434761 10.1016/j.ympev.2007.02.027

